# Links Between Arts and Health, Examples From Quantitative Intervention Evaluations

**DOI:** 10.3389/fpsyg.2021.742032

**Published:** 2021-12-14

**Authors:** Töres Theorell

**Affiliations:** Karolinska Institutet, previous director of the National Institute for Psychosocial Factors and Health, Stockholm, Sweden

**Keywords:** visual arts, music, poetry, leadership, elderly, regenerative hormones, choir singing, school

## Abstract

The author presents eight of his own group’s studies. They have been published from early 1980s until 2016. Each study will be placed in its scientific context and discussed in relation to possible progress in arts and health research. In these examples, statistical methods with longitudinal designs and mostly control groups have been used. Some of them are randomized controlled trials. Physiological and endocrinological variables have been assessed in some of these studies in efforts to increase our understanding of how music experiences and other kinds of arts experiences interact with bodily reactions of relevance for health development. Although some of the studies have suffered from low statistical power and other methodological weaknesses, they show that it is possible to do statistical evaluations of arts interventions aiming at improved health.

## Introduction

The potential of arts intervention both in treatments and in public health work is the main theme of this whole special collection on arts and health. This presentation is focused on examples of research from one group of researchers associated with the National Institute for Psychosocial Factors and Health as well as the Department of Public Health at the Karolinska Institute in Stockholm, Sweden. As the leader of the group, I had a background working as a physician in cardiology, general practice, and social medicine. The studies described here took place during three decades, starting from the early 1980s. This reports a summary presentation of eight studies. The aim is to show how the studies were framed and how the scientific background influenced the design and execution of the studies. The examples illustrate how contemporary developments in basic science have influenced the choice of assessments in studies of health effects of arts interventions. They also show that the field has had poor financial support in Sweden. This has resulted in small-scale studies. Since interest in the research area across the Nordic region is increasing, this is likely to change.

## Description of the Studies

Tables 1–3 show an overview of the eight evaluation studies.

**Table 1:** Study characteristics.

Study name	Year and authors	Design	Sample sizes
Arts activation for elderly	*Arnetz et al., 1983*	Intervention and comparable contr	Matched age, sex, 30/30
Visual arts for elderly women	(Wikström et al., 1993)	Random allocation to intervention/talk	Matched pairs 20/20
Choir singing and IBS	Grape et al., 2009, 2010	Random allocation to choir/talk	No prev choir 28/27->13/14
Dance therapy for fibromyalgia	Bojner Horwitz et al., 2003	Random allocation to dance/usual care	20(oversampl) vs. 16
Multiple arts for burnout	Grape Viding et al., 2015	Random allocation to arts/usual care	36(oversampl) vs. 12
Weekly joy music against school stress	Lindblad et al., 2007	Comparable groups music/computer/usual	16/18/64 ->13/15718
Art psychotherapy in psychosomatics	Theorell et al., 2021	Referred patients with long-term illness	24 patients with full partic.
Art-based leadership training	Romanowska et al., 2011, 2016	Random allocation of matched managers with subordinates	18/19 manag. 41/58 subord. 55/66% partic.

**Table 2:** Interventions.

Study name	Intervention description
Arts activation for elderly	Exploration of previous individual arts activities and formation of active culture groups (interv) or usual routine (contr.) 6months
Visual arts for elderly women	Discussion based on inspection of fine art (contr) or daily events (contr.) 4months plus follow-up 4months
Choir singing and IBS	Choir singing once/week (interv) or lecture plus discussion 1/w (contr.) for 1year
Dance therapy for fibromyalgia	Weekly dance therapy (interv) for 6months or usual care (cont) for 6months with 8 months follow-up
Multiple arts for burnout	Every week rotating art intervention twice/week (interv) for 3months or usual care (cont) for 3months plus 8months follow-up
Weekly joy music against school stress	One hour extra social music/week (interv) or extra computer learning (contr 1) or usual curriculum (contr 2) for one school year
Art psychotherapy in psychosomatics	Long-term psychosomatic patients referred for art psychotherapy for up to 2years
Art-based leadership training	Poems and music with group discussions (interv) vs. lectures without arts (contr)

**Table 3:** Observations and results.

Study name	Observation periods	Significant results
Arts activation for elderly	Pre and post 3months and 6months of intervention	Improved emotions, social activity and physiology in intervention group
Visual arts for elderly women	Pre and post interv (4months) and follow-up (+4months)	Improved emotions, social activity, and blood pressure in intervention group
Choir singing and IBS	Pre and post 6, 9, and 12months of interventions	First half-year marked testosterone increase, first year improved fibrinogen in choir group
Dance therapy for fibromyalgia	Pre and post interv (6months) and follow-up (+8months)	Improved mobility, less pain and more energy after dance therapy
Multiple arts for burnout	Pre and post interv (3months) and follow-up (+3months)	Improvement of alexithymia and burnout scores in interv group until follow-up
Weekly joy music against school stress	Pre (August), mid school year (December) and end (June)	Decrease in mid-day saliva cortisol in music gr but not in other groups
Art psychotherapy in psychosomatics	Every 4–6months during 2-year weekly therapy	Decreased anxiety, depression after 1year, increased energy, and uric acid post 6months
Art-based leadership training	Pre and post interv (12months) and follow-up (+6months)	After 18months improved mental health, DHEA-s in intervention subordinates

### Arts Activation in Home for Elderly

The first study (Arnetz et al., 1983) was launched during a period in science when techniques for the assessment of endocrine changes were developed. Endocrine pathways had previously been introduced in psychosomatic medicine and stress research (Selye, 1950; von Euler and Lishajko, 1961; Levi, 1965; Frankenhaeuser et al., 1968; Dantzer et al., 1980), but more and more sensitive assessment methodologies were developed (see Dantzer and Kelley, 1989), and it therefore became possible to detect small changes due to subtle psychological reactions. Of particular interest for research in the field of possible relationships between cultural activity and health was that John Mason (1968) had introduced the positive stress-counterbalancing pathway which can be summarized as the hypothalamo-pituitary-gonadal (HPG) axis. According to Mason, the hypothalamo-pituitary-adrenocortical (HPA) axismobilizes energy during challenging situations and is balanced by the restorative and regenerative function of the HPG axis active during rest and joyful stimulation. During such periods hormones which belong to this group, such as sex hormones and their precursors (DeHydro-Epi-Androsterone, DHEA, and DeHydro-Epi-Androsterone sulfate, DHEA-s, in both men and women), stimulate the replacement of worn-out cells and repair damaged cells. The excretion of these hormones can be assessed in blood, urine, and saliva.

In a home for elderly people, one unit (floor in the building) was assigned to be intervention and another one to be the control group. The experimental period lasted for 6months, and assessments were made before start, after 3months, and at the end of the intervention period. Standardized assessments of emotional states, social activity, height and weight, blood pressure and morning blood concentration of HbA1C (reflecting accumulated changes in blood sugar during a couple of weeks) as well as hormones which mirror regenerative (anabolic, HPG) activity and energy mobilization, respectively, were made.

The staff examined every tenant’s previous life experiences of cultural activities and using this information, interest groups were created based on those tenants that turned out to have the same interests. This exploration led to the formation of several cultural groups: one for botany, one for visual arts, one for history, one for instrumental music and one for song. The visual arts group jointly constructed a piece of art, and the history group studied the history of the place during the winter and then made walks in historically interesting surroundings.

There were 30 participants in each group. The two selected units had 60 tenants each, but the selection of participants was made with the goal of creating two strictly comparable samples with participants who would be able to finish the study. The selection process was made in cooperation with the staff. Non-participation was low, and the “intention to treat” principle was used which means that non-active subjects were included in the evaluation. The samples were matched for age group, sex, and physical and psychological disability.

The results (Arnetz et al., 1983; Arnetz and Theorell, 1983) indicated favorable changes in social activities in the intervention compared to the control group from start to 6months. This increase in improved social activity was paralleled by an increase in regenerative hormone activity (sex hormones and DHEA) and in decreased concentration of HbA1C, which is likely to mirror decreased energy mobilization or “stress.”

This arts activation of elderly accordingly showed promising results which indicated that it may be possible to influence life for institutionalized elderly by means of arts activities resulting in improved hormonal balance. However, several parameters were assessed, and the samples were relatively small. This might have resulted in randomly “significant” findings, but the all-over pattern clearly pointed in a positive direction. It could be argued that a strict individual randomization would have been preferable. However, most of the arts activities performed were collective in nature and changes in the whole ward could be one of the possible beneficial results of the intervention. Thus, the ideal study should have included several wards and much larger samples in a cluster randomized trial.

With the design employed it is impossible to know whether the crucial element is the art stimulation itself or not. It could be that any intervention aiming at increased social activity could have induced similar results. What the results do show is that the package that was used has had clear positive effects. One interpretation is that arts activities could be potent stimuli for togetherness.

Similar studies have not been published but our group has recently used a daily self-selected music listening program for demented home-cared subjects together with their caring relative. This was evaluated by means of repeated saliva assessments of cortisol (representing HPA) and DHEA-s (representing HPG) every day during a 2-month period. The findings showed that one-fourth of the patient-relative couples had decreasing cortisol levels and improved balance between HPA and HPG during the intervention period. For the relatives, a significantly improved circadian pattern was observed (Theorell et al., 2021) on the whole group level. Results from a control group are necessary for the interpretation of results and results from such a group are forthcoming.

After completing this first study the decision was made to take the findings to new studies of elderly to further explore the potential role that more specific cultural activities could play for the health of elderly.

### Visual Arts Stimulation for Elderly Women

The framework of this study (Wikström et al., 1993) was the same as in the first study, a home for elderly where each tenant lives in an apartment of his/her own but joint main meals were organized once a day. Forty-six women living alone with age at least 70 were asked to participate. The potential participants were matched pairwise so that within each pair the subjects were comparable in terms of handicap and age. Within each pair a randomization was performed, with allocation either to art intervention or control condition. Thus, the study differed from the previous one since the formation of groups was based upon individual randomization. The intervention was much less “social” and more limited – looking at pictures of visual fine arts and discussing thoughts evoked by these.

Twenty participants in each groups completed the study with measurements before start, again after 4months (when the intervention had been finished), and were finally followed up 4months later. The intervention took place once every week during the four intervention months and lasted for 1h on each occasion. Attrition was not a problem. The experimental leader (Wikström) carried with her a small projector by means of which she could show pictures of fine art (by artists, such as van Gogh, Monet, and Manet, and Swedish artists, such as Zorn, Larsson, and Sandberg) with defined themes, such as “playing children,” “flowers,” and “women.” In each session, pictures illustrating one of these themes were shown. After each picture, there was a discussion regarding feelings and thoughts evoked by the picture. In the control group the experimental leader spent the same amount of time once a week for 4months discussing societal events that had occurred during the past days.

Statistically significant findings (two-way interactions) in this study comparing the development in the two groups showed that the women in the arts group, but not in the control group, developed more joyful and less depressed feelings, decreased systolic blood pressure, and an improved ability to interpret visual presentations according to a standardized test. An interesting observation was that the consumption of laxatives decreased in the art group but remained constant in the control group. Improvements in the intervention group stayed significant after the four-month follow-up after the end of the interventions.

This study indicates that repeated conversations based on thoughts evoked by fine arts stimulation may have stronger effects on well-being among elderly institutionalized women than conversation about daily events reported in newspapers. The finding contributes to the question whether arts interventions add anything that cannot be achieved by conversations or social contact in general.

A critique of the study design is that the experimenter performed the conversations in both groups, and hence, bias cannot be ruled out. There was awareness of this methodological problem, and the experimenter accordingly made efforts to make both interventions interesting and intellectually rewarding for the participants. Another critique is that many statistical comparisons were performed on two relatively small groups with possible random significance. However, the findings follow a meaningful pattern. No strictly comparable study has been published elsewhere, but art therapy (including both active and passive components) has been used for instance in breast cancer rehabilitation therapy, and it has been shown in a randomized controlled study to improve coping resources in cancer patients (Öster et al., 2006).

After having devoted most of our energy in the first studies to elderly, we turned to younger subjects in further work and focused on a specific group with a disorder that is according to most researchers partly psychosomatic (Hausteiner-Wiehle and Henningsen, 2014). The underlying idea is that psychosomatic patients represent a sensitive group who could be hypothesized to display more obvious effects of arts intervention than others. In this case, we also used a more specific arts intervention, namely choir singing.

### Choir Singing and Irritable Bowel Syndrome

In population studies, irritable bowel syndrome (IBS) has been shown to be a problem for up to 10% of the normal adult population. Among etiological factors diet, circadian meal rhythmicity, and psychological stress have been mentioned. It has been shown (Jonsson and Hellström, 2000) that the blood concentration of a hormone, motilin, that regulates peristalsis in the gut, is disturbed by provocation of thoughts about stressful life experiences.

Given the evidence that singing can relieve stress (Clift et al., 2008, 2009), this study explored the idea that choir singing could be beneficial for IBS patients. Patients were recruited *via* advertisements in daily newspapers and from the union for patients with gastrointestinal disorders.

After screening, 28 subjects were randomly allocated to the choir and 27 to the control group (Grape et al., 2009, 2010). The choir participants met for choir singing once a week during a school year (autumn and spring semester) while the participants in the control group met for lectures about IBS with approximately the same frequency during the same period. Choice of music for the choir rehearsals was adapted to the fact that most participants had no experience of choir singing. The choir year ended in a performance for the control group. In the control group, the participants listened to short lectures about various recommendations for IBS patients and had group discussions regarding what had been said (“information group”). Questionnaires and blood samples were collected before as well as after 6, 9, and 12months in both groups. On the same days, saliva samples were collected from each subject during waking hours of the day on six occasions from awakening to bedtime. After 12months, 13 subjects remained in the intervention and 14 subjects in the control group (46 and 52%, respectively). Thus, although there was approximately 50% attrition altogether, the drop-out frequency was comparable in the two groups. An intervention of this intensity (once a week) over a whole year in a community intervention is likely to be associated with high attrition.

The one-year findings showed borderline significant findings (*p*=0.08) for motilin – the hormone regulating gut activity mentioned above – and self-reported IBS pain according to scores in the standardized questionnaire (p=0.08). There was a significant interaction, however, in two-way analysis of variance (*p*=0.047) for plasma concentration of fibrinogen which increased in the control group but remained stable in the choir group. An interpretation is that the participation in choir singing may have prevented an adverse increase in inflammatory activity observed in the control group. Fibrinogen mirrors pro-inflammatory activity. It also has an important role in coagulation – increasing fibrinogen is associated with increasing tendency to clot formation and increased velocity of atherosclerosis. All these findings were in the direction of a more beneficial development during the study year in the choir group. From start to 6months, there was a significant rise in saliva testosterone concentration in the choir group (+70%) but no such change in the information group (−20%) with a significant group-time interaction (*p*=0.01). This testosterone difference between the groups only lasted during the first half-year and was not maintained until the end of the year. Testosterone stimulates regeneration (see above, HPG axis) in all cell systems in the body (see Theorell, 2016). In normal physiological regulation, both in men and in women, an increase in saliva testosterone corresponds to improved health and increased regeneration of worn-out tissues. This serves as protection against adverse effects of stress. We could only speculate about reasons for the relatively short duration of this positive hormonal effect.

Most of the findings were only tentative but one interpretation is that choir singing may induce more beneficial physiological processes in this group of patients than conversations/lectures without singing or other components of arts.

### Dance Therapy for Fibromyalgia Patients

Another specific art form is dancing. It is frequently argued that dance and music are closely related arts activities and that it would be logical to study possible effects of dance therapy in psychosomatic conditions. The decision was to focus on another severe psychosomatic condition, fibromyalgia, which was a relatively common diagnosis in Sweden at the time (Lindell et al., 2000). Fibromyalgia patients suffer from extreme fatigue and muscle pain.

The fibromyalgia dance study (Bojner Horwitz et al., 2003) involved patients who were recruited from rheumatology specialists in Stockholm. Thirty-six patients with an established diagnosis were randomly allocated either to treatment with dance therapy once a week or to “usual therapy” for 6months. There was deliberate oversampling for the intervention group (20 versus 16 subjects). Assessments were made before the start of dance therapy, immediately after the therapy period 6months later, and then again after a follow-up period of 8months. Attrition was not a problem.

Standardized ratings of pain, mobility, and energy were made as well as morning sampling of saliva and blood for the assessment of stress-related hormones. Video recordings of standard movements performed by the patients were made in conjunction with the assessments (Bojner Horwitz et al., 2004).

The results 14months after start showed statistically significantly improved self-ratings of pain, energy, and mobility in the dance group but not in the control group. Interestingly, when patients had inspected their own video recordings of standard movements on the same occasions, they rated their own improvements as more convincing. No significant changes were found for the stress hormone measures.

Research on possible relationships between dance and health is growing. Recently, research (Bojner Horwitz et al., 2015) has shown that the development of dancing skill is associated with greater ability to communicate feelings. A randomized controlled intervention trial showed that adolescent girls with mild psychosomatic symptoms showed improved subjective health from weekly free dance activity but also that the intervention must be sustained for long periods (Duberg et al., 2013). Thus, the relevance of dance therapy has been strengthened by later research.

The next step in our efforts to throw light on possible effects of arts activities on health was to apply a multi-cultural approach in our intervention. Our focus was on exhaustion syndrome (burnout) which had at the time replaced fibromyalgia as the most frequently diagnosed severe long-lasting condition resulting in work absence (Höglund et al., 2020). Exhaustion syndrome is characterized as a stress-related condition with chronic fatigue. Cognitive difficulties are common.

### Palette Study – Multiple Arts for Women With Burnout Syndrome

The Palette study aimed to evaluate the effect of exposure to multiple art forms for patients with burnout syndrome (Grape Viding et al., 2015). Neurobiological research had shown that different areas of the brain are stimulated by visual art and by music but also that the intensity of the reaction in both types of areas was stronger when visual and musical stimulation were presented simultaneously (Baumgartner et al., 2006). This was an important observation because it showed that concomitant exposure to several kinds of arts activities might have stronger effects than interventions with only one kind of arts activity. Later research has shown (Lennartsson et al., 2017) that expertise in music as well as in other kinds of fine arts (writing, visual arts, and theater) is associated with good ability to handle emotions and that concomitant competence in several kinds of arts is associated with even better emotional ability.

The Palette study was based on the researcher group’s assumption that alexithymia is an important factor in the burnout syndrome (Grape Viding et al., 2015). Alexithymia is lack of ability to differentiate, verbalize, and communicate feelings. When subjects lack these abilities, they have difficulties to handle stressful situations and this may result in sleep problems and long-lasting lack of regeneration, which induces a state of increased psychological and somatic vulnerability. In the intervention group, the participants had a rotating exposure to many kinds of cultural activity (two occasions for each participant of interactive theater, movie, vocal improvisation and drawing, dance, mindfulness training, and musical show) once a week over a period of 3months. This differs from the arts activation for elderly people described above since the activities in that study were self-selected on a group basis and the participants stayed in the group throughout the intervention period. These two strategies could be described as “rotating” and “non-rotating” interventions. In the control group in the Palette study, the participants had health care center routine care. Both programs were housed in the patients´ own health care centers. Attrition was not a problem.

After the diagnosis of burnout syndrome had been established, 55 female patients were recruited in the three health care centers. Seven were excluded because they had serious depression. The remaining 48 patients were randomly allocated to the intervention and control group, respectively, with deliberate oversampling to the intervention group, 36 in the intervention and 12 in the control group. Using standardized questionnaires, assessments were made of degree of exhaustion syndrome and alexithymia before start, at the end of the intervention period, and finally after the additional 3months of follow-up. The results showed a statistically significant improvement both in alexithymia and exhaustion syndrome scores in the intervention group but not in the control group. This difference was sustained in the follow-up assessments.

The results supported the hypothesis that 3months of “rotating” multi-cultural intervention would be associated with decreased emotional exhaustion and alexithymia. It was positive that the attrition was small. Again, in this kind of intervention it is impossible to know what components of the intervention are effective. What can be concluded is that a “package” of cultural experiences may have beneficial effects.

### Joyful Music for Pupils and Lowered Stress Levels in the Classroom

This study (Lindblad et al., 2007) was on the ancient idea that music has the ability of increasing “togetherness” if it is “distributed” in a constructive way. The importance of this was confirmed by Spychiger (1995) who performed a large music intervention study of 52 classes in Switzerland representing different ages in the Swiss compulsory school system. Half of the classes were randomly assigned an extra hour of (social) music lessons per week while the other half followed the “regular curriculum” group. The intervention and the evaluation assessments in both groups lasted for 3years. The major finding was that the social skills in the pupils improved much more over the study period in the music intervention group than in the other group. Similar results were found several years later in a Finnish study of 735 school children aged 9–12. The results showed that the school environment improved for these children and that such an improvement was not observed in a control population (Eerola and Eerola, 2014).

In our own study (Lindblad et al., 2007), a music teacher who had developed a technique for using classroom music as a play tool, aiming at improved cohesiveness among the pupils, distributed the intervention program which took place in the classroom for an hour once every week during a school year. The informal title of the project was: “By doing fun music together we discover one another.” Pupils in the 5th and 6th school year were randomly assigned to three groups, the music intervention group, an “extra computer training” group and a “regular curriculum” group. There were 17 fully participating subjects in each group. Attrition was not a problem in the music intervention and the extra computer control group but much larger in the regular curriculum control group – in which subjects only responded to questionnaires and delivered saliva samples. Assessments of psychosocial functions and cortisol concentration in saliva samples were made on participants at start of Fall, before Christmas and finally in June before the Summer vacation. The saliva samples were collected at awakening, at mid-day, and finally at bedtime (Lindblad et al., 2007). The results showed that in saliva the mid-day cortisol levels decreased significantly in the music group during the study year (from start to end of year) but not in the other groups. The interpretation is that the atmosphere slowly calmed down during the school year in the music group. The psychosocial assessments, however, did not show any significant change. The study had relatively low statistical power. As with the other studies described here, the classroom-study needs replication.

### Art Psychotherapy for Psychosomatic Patients

There is a growing scientific literature indicating that various forms of therapy utilizing experiences of arts (music therapy, visual arts therapy, dance therapy, and psychodrama) can be of benefit for patients with long-lasting stress-related conditions, such as sleep disorder (Feng et al., 2018), chronic pain (Lee, 2016), and depression (Tang et al., 2020). In the present study, patients with long-lasting psychosomatic conditions resulting in partial or total loss of working capacity for at least 1year (Theorell et al., 1998) were treated in the “art psychotherapy” program. All the patients had chronic pain conditions but most of them also other illnesses, such as high blood pressure and gastrointestinal disorders. The patients were referred to the program from a rehabilitation center. They were informed that the expected duration of art psychotherapy treatment was 2years. After careful psychosomatic examination each patient was allocated to treatments once a week for 2years in either visual art therapy, music therapy, dance therapy or psychodrama. The kind of art therapy judged to be best for the patient was selected by the total group of therapists (psychologist, physician, visual art therapist, music therapist, dance therapist, and psychodrama expert). The therapists had monthly follow-up meetings in a collaborative effort to use mutual competence. For instance, dramatic memories during a music therapy session may have been discussed during such a therapy meeting. The music therapist may have been advised to stimulate the patient to draw or paint this memory, and then, the resulting picture was interpreted by the therapist group on a subsequent meeting.

Referring to the terminology presented above regarding multi-arts interventions, this program was “non-rotating,” but occasionally input from the other forms of therapy was used.

Three-fourths of those who started stayed in treatment as long as the therapist considered it optimal. Twenty-four participants (22 women and two men) had their treatment started on average 2years (range 13–42months) before the end of the treatment period and participated in the evaluations. At 4–6-month intervals, blood samples were drawn for the assessment of serum uric acid (a proxy measure for energy level, the higher the uric acid level the higher the energy level) and the regenerative hormone DHEA-s. On every assessment occasion, an exploration of psychological state (anxiety and depression) was also performed by means of self-administered standardized questionnaires.

The results indicated that the first year of treatment was characterized by emotional turmoil paralleled by increased energy level (starting from sub-normal level) reflected in temporary significant elevation of serum uric acid. The regenerative hormone DHEA-s had low concentration in the group and did not improve during the study period. Significant improvement was observed in anxiety and depression after 1year of treatment. A tendency toward decreased levels of somatic symptoms in general was observed after 2years of treatment. One-fourth of the patients increased their working activity.

A major weakness in this study was the absence of a control group. It should be pointed out, however, that the chronic conditions that the patients were suffering from had lasted for at least a year and that spontaneous recovery could not be expected. The improvements were slow, and evidence of increased regenerative hormone activity was not found. No similar studies have been published which could confirm or reject these results.

The final report describes a different target group, It was explored whether it would be possible to establish health effects in a “third part.” A framework for improvement of social and emotional manager skills was established. It was examined whether a combined arts intervention (music and poetry) triggering ethical group discussions could be superior in improving social skills among participating managers in comparison with an established cognitively framed management course. Even more importantly, it was explored whether this might lead to improved health among employees. An RCT design was used.

### Art-Based Leadership Training

No scientific evaluations have been published on the potential for beneficial personnel health effects of efforts to influence managers in the direction of increased emotional engagement in their staff by means of arts intervention (Romanowska et al., 2011, 2016). An arts intervention program for this purpose was created. Carefully selected poems dealing with ethically provoking questions with relevance for managers were read professionally to the whole intervention group of managers. The messages were accompanied by specific recorded music. After the performances, there were group discussions regarding the contents. Participating managers met for these sessions once a month for 9months. They also wrote diaries between the meetings and their diary thoughts were used in discussions.

The control intervention program consisted of a widely accepted manager competence training program with the same amount of time engagement, lectures with cognitive contents and group discussions but no artistic components, also once a month over 9months.

Fifty managers were randomly allocated to either arts or control program. Assessments were made before the start, at 1year, and finally 18months later. For each participating manager three subordinates were selected for parallel assessments in both groups. Attrition was a problem due to the long study period since both managers and subordinates in some cases moved to other workplaces, and when a manager moved, data from the corresponding subordinates could not be used. Only one manager in the intervention program and two managers in the control program were lost due to resistance against the respective program, however. Participants in the final analyses on all three occasions were around 20 in both groups for the managers and 35 and 55, respectively, for the subordinates. Standardized questionnaires for the assessment of psychosocial variables and analyses of the hormone parameters serum cortisol and DHEA-s were used on all occasions.

After 18months a statistically significant advantage was observed for the art-based group: In particular, in the art group subordinates compared to those in the conventional group, there was significantly more improvement of mental health and constructive coping, as well as reduced performance-based self-esteem. In addition, there was significantly less of the expected winter/fall deterioration in the serum concentration of the regenerative/anabolic hormone dehydroepiandrosterone-sulfate, but no significant changes were found for cortisol.

## Discussion

The eight studies described above illustrate that it is possible to do quantitative evaluation studies using statistical analyses of health effects of arts interventions. Most of them have had relatively small statistical power, but despite this it has been possible to show statistically significant health effects. All of them need to be replicated using larger samples, and a more rigorous statistical design with pre-calculation of sample sizes is needed.

In several of our studies there have been statistical tests of many outcome variables – which means that there may have been risk of random “mass” significances. However, in those studies there have been significant effects in several domains (psychosocial as well as biological) that all point in the same direction. Both psychosocial and physiological variables have shown significant changes in the expected positive health direction. One important aspect of the multi-domain assessments in these studies is that it allows “triangulation.” For instance, if there are positive effects of an arts intervention on regenerative hormones and at the same time positive mood changes and improved social activity, these observations mutually support one another.

Many different methodologies have been used, and it is impossible to present all of them here, due to limited space. The reader is referred to the original publications where the methods are described in detail. In general, however, we have used endocrinological and physiological measures as well as standardized questionnaires, such as measurements of mood and social activities constructed for more general purposes. Accordingly, no measures have been constructed specifically for these studies. This is intentional since we want to make our results comparable to those obtained in other intervention studies.

The way in which participants were recruited merits some discussion. Before randomization takes place, it is important that participants have been fully informed about the conditions of the study. This is one way of avoiding *differential attrition*. It is always important to bear in mind that the recruitment of participants may limit the generalizability of the findings. In the leadership study, we were very careful in designing the study in such a way that we would avoid differential attrition, and we were successful in this. However, in the preparatory stages many potential participants were contacted who were not willing to accept the conditions and therefore declined to take part. Accordingly, the two study groups were comparable and the differences between them reliable. But on the other hand, the participants in *both* groups were more willing to accept randomization and repeated data collection than managers in general.

Several other groups of researchers have examined various aspects of the biological effects of arts experiences. For instance, the effects of music listening on hormones and other chemical agents in the body (blood and saliva) have recently been reviewed by Finn and Fancourt (2018) who arrive at similar conclusions to ours regarding needs for stricter research designs. During our years in this research field, on a broader scale there has been a growing interest in the arts and health research field. This has recently been summarized by Fancourt and Finn (2019). The experience in our group has been that participants (from youngsters to elderly) are mostly willing to accept physiological and endocrinological measures and that arts interventions often show significant effects in such domains. This is of great help in our wholistic understanding of health effects of arts interventions. Careful measuring is often expensive, however. In our most recent study of subjects with dementia and their closest relative (Theorell et al., 2021), saliva hormone levels were measured daily both in mornings and evenings for 2months and this was costly to undertake.

One example of more specific research is the organized choir singing for patients with lung disorders, such as chronic obstructive lung disease. A consensus has been reached (Lewis et al., 2016) that regular participation in choir singing for this group has beneficial effects on quality of life and that this helps patients cope with their difficult life situation although the studies also show that choir singing does not improve objectively measured lung function.

A similar research area is the health effects of choir singing for patients with mental disorders. Williams et al. (2018) recently showed in a systematic review that choir singing benefits quality of life for that group as well. Another widely accepted use of arts stimulation is dance and music therapy for patients with Parkinson’s disease. A recent review (Pereira et al., 2019) was based upon five reviews and 40 experimental papers. The conclusion was that rhythmic stimulation and dance provide motor, cognitive, and quality of life benefits for participants with Parkinson’s disease. Sound stimuli and dance offer measurable effects on gait and favorably affect cognitive abilities.

An important background variable that should be taken into account is genetic background. In a recent study of twins aged 27–54, we have shown that there is a statistically significant relationship between hours of lifelong practice of piano playing and ability to handle emotions (Theorell et al., 2014). This relationship, however, is pleiotropic. Ability to handle emotions and willingness to practice piano playing are both partly genetically determined, and when these relationships are combined in one model, the genetic background explains the statistical relationship between many hours of music practicing and good emotional ability. Still, cultural interventions can favorably affect ability to handle emotions, as shown for example in study 5. Accordingly, we need to take into account gene–environment interactions. One practical aspect of this is that intervention evaluations should preferably be based on large samples.

Among several outcome measures used in this research, regenerative hormones (testosterone and DHEA-s) have played an important role. Is there any stimulative effect of arts experiences on the excretion of regenerative hormones? Is so, this could be a link between cultural activities and health in a broad sense. Among the five studies that have included these measures, two were studies of long-lasting chronic conditions (fibromyalgia and long-lasting psychosomatic conditions), whereas three were studies of less serious conditions (normal ageing, irritable bowel syndrome and working managers with their employees). It was observed that the interventions did not have any significant regeneration effects in the chronic conditions whereas there were observable effects in the more “normal” groups. This is also an observation that needs follow-up.

The studies point at possible practical applications for primary schools, psychosomatic care, and care of elderly as well as programs for improved management. But as was pointed out by Fancourt and Finn (2019) there are numerous systematic reviews indicating other potential applications. It is time for larger rigorous evaluation efforts on a societal level.

## Author Contributions

The author confirms being the sole contributor of this work and has approved it for publication.

## Funding

This is a review of several projects with several funding agencies.

## Conflict of Interest

The author declares that the research was conducted in the absence of any commercial or financial relationships that could be construed as a potential conflict of interest.

## Publisher’s Note

All claims expressed in this article are solely those of the authors and do not necessarily represent those of their affiliated organizations, or those of the publisher, the editors and the reviewers. Any product that may be evaluated in this article, or claim that may be made by its manufacturer, is not guaranteed or endorsed by the publisher.
